# Search for long-lived particles decaying to jet pairs

**DOI:** 10.1140/epjc/s10052-015-3344-6

**Published:** 2015-04-17

**Authors:** R. Aaij, B. Adeva, M. Adinolfi, A. Affolder, Z. Ajaltouni, S. Akar, J. Albrecht, F. Alessio, M. Alexander, S. Ali, G. Alkhazov, P. Alvarez Cartelle, A. A. Alves, S. Amato, S. Amerio, Y. Amhis, L. An, L. Anderlini, J. Anderson, R. Andreassen, M. Andreotti, J. E. Andrews, R. B. Appleby, O. Aquines Gutierrez, F. Archilli, A. Artamonov, M. Artuso, E. Aslanides, G. Auriemma, M. Baalouch, S. Bachmann, J. J. Back, A. Badalov, C. Baesso, W. Baldini, R. J. Barlow, C. Barschel, S. Barsuk, W. Barter, V. Batozskaya, V. Battista, A. Bay, L. Beaucourt, J. Beddow, F. Bedeschi, I. Bediaga, S. Belogurov, K. Belous, I. Belyaev, E. Ben-Haim, G. Bencivenni, S. Benson, J. Benton, A. Berezhnoy, R. Bernet, A. Bertolin, M.-O. Bettler, M. van Beuzekom, A. Bien, S. Bifani, T. Bird, A. Bizzeti, P. M. Bjørnstad, T. Blake, F. Blanc, J. Blouw, S. Blusk, V. Bocci, A. Bondar, N. Bondar, W. Bonivento, S. Borghi, A. Borgia, M. Borsato, T. J. V. Bowcock, E. Bowen, C. Bozzi, D. Brett, M. Britsch, T. Britton, J. Brodzicka, N. H. Brook, A. Bursche, J. Buytaert, S. Cadeddu, R. Calabrese, M. Calvi, M. Calvo Gomez, P. Campana, D. Campora Perez, L. Capriotti, A. Carbone, G. Carboni, R. Cardinale, A. Cardini, L. Carson, K. Carvalho Akiba, RCM Casanova Mohr, G. Casse, L. Cassina, L. Castillo Garcia, M. Cattaneo, Ch. Cauet, R. Cenci, M. Charles, Ph. Charpentier, M. Chefdeville, S. Chen, S.-F. Cheung, N. Chiapolini, M. Chrzaszcz, X. Cid Vidal, G. Ciezarek, P. E. L. Clarke, M. Clemencic, H. V. Cliff, J. Closier, V. Coco, J. Cogan, E. Cogneras, V. Cogoni, L. Cojocariu, G. Collazuol, P. Collins, A. Comerma-Montells, A. Contu, A. Cook, M. Coombes, S. Coquereau, G. Corti, M. Corvo, I. Counts, B. Couturier, G. A. Cowan, D. C. Craik, A.C. Crocombe, M. Cruz Torres, S. Cunliffe, R. Currie, C. D’Ambrosio, J. Dalseno, P. David, P. N. Y. David, A. Davis, K. De Bruyn, S. De Capua, M. De Cian, J. M. De Miranda, L. De Paula, W. De Silva, P. De Simone, C.-T. Dean, D. Decamp, M. Deckenhoff, L. Del Buono, N. Déléage, D. Derkach, O. Deschamps, F. Dettori, B. Dey, A. Di Canto, A Di Domenico, H. Dijkstra, S. Donleavy, F. Dordei, M. Dorigo, A. Dosil Suárez, D. Dossett, A. Dovbnya, K. Dreimanis, G. Dujany, F. Dupertuis, P. Durante, R. Dzhelyadin, A. Dziurda, A. Dzyuba, S. Easo, U. Egede, V. Egorychev, S. Eidelman, S. Eisenhardt, U. Eitschberger, R. Ekelhof, L. Eklund, I. El Rifai, Ch. Elsasser, S. Ely, S. Esen, H.-M. Evans, T. Evans, A. Falabella, C. Färber, C. Farinelli, N. Farley, S. Farry, R. Fay, D. Ferguson, V. Fernandez Albor, F. Ferreira Rodrigues, M. Ferro-Luzzi, S. Filippov, M. Fiore, M. Fiorini, M. Firlej, C. Fitzpatrick, T. Fiutowski, P. Fol, M. Fontana, F. Fontanelli, R. Forty, O. Francisco, M. Frank, C. Frei, M. Frosini, J. Fu, E. Furfaro, A. Gallas Torreira, D. Galli, S. Gallorini, S. Gambetta, M. Gandelman, P. Gandini, Y. Gao, J. García Pardiñas, J. Garofoli, J. Garra Tico, L. Garrido, D. Gascon, C. Gaspar, U. Gastaldi, R. Gauld, L. Gavardi, G. Gazzoni, A. Geraci, E. Gersabeck, M. Gersabeck, T. Gershon, Ph. Ghez, A. Gianelle, S. Gianì, V. Gibson, L. Giubega, V. V. Gligorov, C. Göbel, D. Golubkov, A. Golutvin, A. Gomes, C. Gotti, M. Grabalosa Gándara, R. Graciani Diaz, L. A. Granado Cardoso, E. Graugés, E. Graverini, G. Graziani, A. Grecu, E. Greening, S. Gregson, P. Griffith, L. Grillo, O. Grünberg, B. Gui, E. Gushchin, Yu. Guz, T. Gys, C. Hadjivasiliou, G. Haefeli, C. Haen, S. C. Haines, S. Hall, B. Hamilton, T. Hampson, X. Han, S. Hansmann-Menzemer, N. Harnew, S. T. Harnew, J. Harrison, J. He, T. Head, V. Heijne, K. Hennessy, P. Henrard, L. Henry, J. A. Hernando Morata, E. van Herwijnen, M. Heß, A. Hicheur, D. Hill, M. Hoballah, C. Hombach, W. Hulsbergen, N. Hussain, D. Hutchcroft, D. Hynds, M. Idzik, P. Ilten, R. Jacobsson, A. Jaeger, J. Jalocha, E. Jans, A. Jawahery, F. Jing, M. John, D. Johnson, C. R. Jones, C. Joram, B. Jost, N. Jurik, S. Kandybei, W. Kanso, M. Karacson, T. M. Karbach, S. Karodia, M. Kelsey, I. R. Kenyon, T. Ketel, B. Khanji, C. Khurewathanakul, S. Klaver, K. Klimaszewski, O. Kochebina, M. Kolpin, I. Komarov, R. F. Koopman, P. Koppenburg, M. Korolev, L. Kravchuk, K. Kreplin, M. Kreps, G. Krocker, P. Krokovny, F. Kruse, W. Kucewicz, M. Kucharczyk, V. Kudryavtsev, K. Kurek, T. Kvaratskheliya, V. N. La Thi, D. Lacarrere, G. Lafferty, A. Lai, D. Lambert, R. W. Lambert, G. Lanfranchi, C. Langenbruch, B. Langhans, T. Latham, C. Lazzeroni, R. Le Gac, J. van Leerdam, J.-P. Lees, R. Lefèvre, A. Leflat, J. Lefrançois, O. Leroy, T. Lesiak, B. Leverington, Y. Li, T. Likhomanenko, M. Liles, R. Lindner, C. Linn, F. Lionetto, B. Liu, S. Lohn, I. Longstaff, J. H. Lopes, P. Lowdon, D. Lucchesi, H. Luo, A. Lupato, E. Luppi, O. Lupton, F. Machefert, I. V. Machikhiliyan, F. Maciuc, O. Maev, S. Malde, A. Malinin, G. Manca, G. Mancinelli, A. Mapelli, J. Maratas, J.F. Marchand, U. Marconi, C. Marin Benito, P. Marino, R. Märki, J. Marks, G. Martellotti, M. Martinelli, D. Martinez Santos, F. Martinez Vidal, D. Martins Tostes, A. Massafferri, R. Matev, Z. Mathe, C. Matteuzzi, A. Mazurov, M. McCann, J. McCarthy, A. McNab, R. McNulty, B. McSkelly, B. Meadows, F. Meier, M. Meissner, M. Merk, D. A. Milanes, M.-N. Minard, N. Moggi, J. Molina Rodriguez, S. Monteil, M. Morandin, P. Morawski, A. Mordà, M. J. Morello, J. Moron, A.-B. Morris, R. Mountain, F. Muheim, K. Müller, M. Mussini, B. Muster, P. Naik, T. Nakada, R. Nandakumar, I. Nasteva, M. Needham, N. Neri, S. Neubert, N. Neufeld, M. Neuner, A. D. Nguyen, T. D. Nguyen, C. Nguyen-Mau, M. Nicol, V. Niess, R. Niet, N. Nikitin, T. Nikodem, A. Novoselov, D. P. O’Hanlon, A. Oblakowska-Mucha, V. Obraztsov, S. Ogilvy, O. Okhrimenko, R. Oldeman, C. J. G. Onderwater, M. Orlandea, B. Osorio Rodrigues, J. M. Otalora Goicochea, A. Otto, P. Owen, A. Oyanguren, B. K. Pal, A. Palano, F. Palombo, M. Palutan, J. Panman, A. Papanestis, M. Pappagallo, L. L. Pappalardo, C. Parkes, C. J. Parkinson, G. Passaleva, G. D. Patel, M. Patel, C. Patrignani, A. Pearce, A. Pellegrino, G. Penso, M. Pepe Altarelli, S. Perazzini, P. Perret, L. Pescatore, E. Pesen, K. Petridis, A. Petrolini, E. Picatoste Olloqui, B. Pietrzyk, T. Pilař, D. Pinci, A. Pistone, S. Playfer, M. Plo Casasus, F. Polci, A. Poluektov, I. Polyakov, E. Polycarpo, A. Popov, D. Popov, B. Popovici, C. Potterat, E. Price, J.D. Price, J. Prisciandaro, A. Pritchard, C. Prouve, V. Pugatch, A. Puig Navarro, G. Punzi, W. Qian, B. Rachwal, J. H. Rademacker, B. Rakotomiaramanana, M. Rama, M. S. Rangel, I. Raniuk, N. Rauschmayr, G. Raven, F. Redi, S. Reichert, M. M. Reid, A. C. dos Reis, S. Ricciardi, S. Richards, M. Rihl, K. Rinnert, V. Rives Molina, P. Robbe, A. B. Rodrigues, E. Rodrigues, P. Rodriguez Perez, S. Roiser, V. Romanovsky, A. Romero Vidal, M. Rotondo, J. Rouvinet, T. Ruf, H. Ruiz, P. Ruiz Valls, J. J. Saborido Silva, N. Sagidova, P. Sail, B. Saitta, V. Salustino Guimaraes, C. Sanchez Mayordomo, B. Sanmartin Sedes, R. Santacesaria, C. Santamarina Rios, E. Santovetti, A. Sarti, C. Satriano, A. Satta, D.M. Saunders, D. Savrina, M. Schiller, H. Schindler, M. Schlupp, M. Schmelling, B. Schmidt, O. Schneider, A. Schopper, M.-H. Schune, R. Schwemmer, B. Sciascia, A. Sciubba, A. Semennikov, I. Sepp, N. Serra, J. Serrano, L. Sestini, P. Seyfert, M. Shapkin, I. Shapoval, Y. Shcheglov, T. Shears, L. Shekhtman, V. Shevchenko, A. Shires, R. Silva Coutinho, G. Simi, M. Sirendi, N. Skidmore, I. Skillicorn, T. Skwarnicki, N. A. Smith, E. Smith, E. Smith, J. Smith, M. Smith, H. Snoek, M. D. Sokoloff, F. J. P. Soler, F. Soomro, D. Souza, B. Souza De Paula, B. Spaan, P. Spradlin, S. Sridharan, F. Stagni, M. Stahl, S. Stahl, O. Steinkamp, O. Stenyakin, F Sterpka, S. Stevenson, S. Stoica, S. Stone, B. Storaci, S. Stracka, M. Straticiuc, U. Straumann, R. Stroili, L. Sun, W. Sutcliffe, K. Swientek, S. Swientek, V. Syropoulos, M. Szczekowski, P. Szczypka, T. Szumlak, S. T’Jampens, M. Teklishyn, G. Tellarini, F. Teubert, C. Thomas, E. Thomas, J. van Tilburg, V. Tisserand, M. Tobin, J. Todd, S. Tolk, L. Tomassetti, D. Tonelli, S. Topp-Joergensen, N. Torr, E. Tournefier, S. Tourneur, M. T. Tran, M. Tresch, A. Trisovic, A. Tsaregorodtsev, P. Tsopelas, N. Tuning, M. Ubeda Garcia, A. Ukleja, A. Ustyuzhanin, U. Uwer, C. Vacca, V. Vagnoni, G. Valenti, A. Vallier, R. Vazquez Gomez, P. Vazquez Regueiro, C. Vázquez Sierra, S. Vecchi, J. J. Velthuis, M. Veltri, G. Veneziano, M. Vesterinen, JVVB Viana Barbosa, B. Viaud, D. Vieira, M. Vieites Diaz, X. Vilasis-Cardona, A. Vollhardt, D. Volyanskyy, D. Voong, A. Vorobyev, V. Vorobyev, C. Voß, J. A. de Vries, R. Waldi, C. Wallace, R. Wallace, J. Walsh, S. Wandernoth, J. Wang, D. R. Ward, N. K. Watson, D. Websdale, M. Whitehead, D. Wiedner, G. Wilkinson, M. Wilkinson, M. P. Williams, M. Williams, H.W. Wilschut, F. F. Wilson, J. Wimberley, J. Wishahi, W. Wislicki, M. Witek, G. Wormser, S. A. Wotton, S. Wright, K. Wyllie, Y. Xie, Z. Xing, Z. Xu, Z. Yang, X. Yuan, O. Yushchenko, M. Zangoli, M. Zavertyaev, L. Zhang, W. C. Zhang, Y. Zhang, A. Zhelezov, A. Zhokhov, L. Zhong

**Affiliations:** Centro Brasileiro de Pesquisas Físicas (CBPF), Rio de Janeiro, Brazil; Universidade Federal do Rio de Janeiro (UFRJ), Rio de Janeiro, Brazil; Center for High Energy Physics, Tsinghua University, Beijing, China; LAPP, Université de Savoie, CNRS/IN2P3, Annecy-Le-Vieux, France; Clermont Université, Université Blaise Pascal, CNRS/IN2P3, LPC, Clermont-Ferrand, France; CPPM, Aix-Marseille Université, CNRS/IN2P3, Marseille, France; LAL, Université Paris-Sud, CNRS/IN2P3, Orsay, France; LPNHE, Université Pierre et Marie Curie, Université Paris Diderot, CNRS/IN2P3, Paris, France; Fakultät Physik, Technische Universität Dortmund, Dortmund, Germany; Max-Planck-Institut für Kernphysik (MPIK), Heidelberg, Germany; Physikalisches Institut, Ruprecht-Karls-Universität Heidelberg, Heidelberg, Germany; School of Physics, University College Dublin, Dublin, Ireland; Sezione INFN di Bari, Bari, Italy; Sezione INFN di Bologna, Bologna, Italy; Sezione INFN di Cagliari, Cagliari, Italy; Sezione INFN di Ferrara, Ferrara, Italy; Sezione INFN di Firenze, Florence, Italy; Laboratori Nazionali dell’INFN di Frascati, Frascati, Italy; Sezione INFN di Genova, Genoa, Italy; Sezione INFN di Milano Bicocca, Milan, Italy; Sezione INFN di Milano, Milan, Italy; Sezione INFN di Padova, Padua, Italy; Sezione INFN di Pisa, Pisa, Italy; Sezione INFN di Roma Tor Vergata, Rome, Italy; Sezione INFN di Roma La Sapienza, Rome, Italy; Henryk Niewodniczanski Institute of Nuclear Physics Polish Academy of Sciences, Kraków, Poland; Faculty of Physics and Applied Computer Science, AGH-University of Science and Technology, Kraków, Poland; National Center for Nuclear Research (NCBJ), Warsaw, Poland; Horia Hulubei National Institute of Physics and Nuclear Engineering, Bucharest-Magurele, Romania; Petersburg Nuclear Physics Institute (PNPI), Gatchina, Russia; Institute of Theoretical and Experimental Physics (ITEP), Moscow, Russia; Institute of Nuclear Physics, Moscow State University (SINP MSU), Moscow, Russia; Institute for Nuclear Research of the Russian Academy of Sciences (INR RAN), Moscow, Russia; Budker Institute of Nuclear Physics (SB RAS) and Novosibirsk State University, Novosibirsk, Russia; Institute for High Energy Physics (IHEP), Protvino, Russia; Universitat de Barcelona, Barcelona, Spain; Universidad de Santiago de Compostela, Santiago de Compostela, Spain; European Organization for Nuclear Research (CERN), Geneva, Switzerland; Ecole Polytechnique Fédérale de Lausanne (EPFL), Lausanne, Switzerland; Physik-Institut, Universität Zürich, Zurich, Switzerland; Nikhef National Institute for Subatomic Physics, Amsterdam, The Netherlands; Nikhef National Institute for Subatomic Physics and VU University Amsterdam, Amsterdam, The Netherlands; NSC Kharkiv Institute of Physics and Technology (NSC KIPT), Kharkiv, Ukraine; Institute for Nuclear Research of the National Academy of Sciences (KINR), Kyiv, Ukraine; University of Birmingham, Birmingham, UK; H.H. Wills Physics Laboratory, University of Bristol, Bristol, UK; Cavendish Laboratory, University of Cambridge, Cambridge, UK; Department of Physics, University of Warwick, Coventry, UK; STFC Rutherford Appleton Laboratory, Didcot, UK; School of Physics and Astronomy, University of Edinburgh, Edinburgh, UK; School of Physics and Astronomy, University of Glasgow, Glasgow, UK; Oliver Lodge Laboratory, University of Liverpool, Liverpool, UK; Imperial College London, London, UK; School of Physics and Astronomy, University of Manchester, Manchester, UK; Department of Physics, University of Oxford, Oxford, UK; Massachusetts Institute of Technology, Cambridge, MA USA; University of Cincinnati, Cincinnati, OH USA; University of Maryland, College Park, MD USA; Syracuse University, Syracuse, NY USA; Pontifícia Universidade Católica do Rio de Janeiro (PUC-Rio), Rio de Janeiro, Brazil; Institute of Particle Physics, Central China Normal University, Wuhan, Hubei China; Departamento de Fisica, Universidad Nacional de Colombia, Bogota, Colombia; Institut für Physik, Universität Rostock, Rostock, Germany; National Research Centre Kurchatov Institute, Moscow, Russia; Instituto de Fisica Corpuscular (IFIC), Universitat de Valencia-CSIC, Valencia, Spain; Van Swinderen Institute, University of Groningen, Groningen, The Netherlands; Celal Bayar University, Manisa, Turkey

## Abstract

A search is presented for long-lived particles with a mass between 25 and 50 $\mathrm{GeV}/\mathrm{c}^{2}$ and a lifetime between 1 and 200$\mathrm{\,ps}$ in a sample of proton–proton collisions at a centre-of-mass energy of $\sqrt{s}=7$ TeV, corresponding to an integrated luminosity of 0.62 $\text{ fb }^{-1}$, collected by the LHCb detector. The particles are assumed to be pair-produced by the decay of a standard model-like Higgs boson. The experimental signature of the long-lived particle is a displaced vertex with two associated jets. No excess above the background is observed and limits are set on the production cross-section as a function of the long-lived particle mass and lifetime.

## Introduction

A variety of models for physics beyond the standard model (SM) feature the existence of new massive particles whose coupling to lighter particles is sufficiently small that they are long-lived. If these massive particles decay to SM particles and have a lifetime between approximately 1$\mathrm{\,ps}$ and 1$\mathrm{\,ns}$, characteristic of weak decays, they can be identified by their displaced decay vertex. Examples of such particles are the lightest supersymmetric particle in SUSY models with baryon or lepton number violation [1–4], the next-to-lightest supersymmetric particle in gravity mediated SUSY [5] and the neutral ${\pi _v}$ particle in hidden valley (HV) models with a non-abelian gauge symmetry [6–8]. The latter model is particularly interesting as it predicts that experimental studies have sensitivity to the production of long-lived particles in SM Higgs decays.

This paper reports on a search for ${\pi _v}$ particles, pair-produced in the decay of a SM-like Higgs particle with a mass of 120 ${\mathrm {GeV}/\mathrm{c}^2}$, close to the mass of the scalar bosondiscovered by the ATLAS and CMS experiments [9, 10].^1^ The ${\pi _v}$ candidates are identified by two hadronic jets originating from a displaced vertex. The vertex is required to be displaced from the proton–proton collision axis by more than 0.4 mm and less than 4.8 mm. The lower bound is chosen to reject most of the background from heavy flavour decays. The upper bound ensures that vertices are inside the LHCb beam pipe, which generates a sizeable background of hadronic interaction vertices. The signal is extracted from a fit to the di-jet invariant mass distribution. The analysis is sensitive to a ${\pi _v}$ particle with a mass between 25 and 50 ${\mathrm {GeV}/\mathrm{c}^2}$ and a lifetime between 1 and 200$\mathrm{\,ps}$. The lower boundary on the mass range arises from the requirement to identify two hadronic jets while the upper boundary is mostly due to the geometric acceptance of the LHCb detector.

This analysis uses data collected in proton–proton ($pp$) collisions at a centre-of-mass energy of $\sqrt{s}=7$ TeV. The data correspond to an integrated luminosity of 0.62 $\text{ fb }^{-1}$, collected during the second half of the year 2011 when an analysis-specific trigger selection was implemented. Although similar searches have been reported by the CDF [11], D0 [12], ATLAS [13] and CMS [14] experiments, LHCb has a unique coverage for long-lived particles with relatively small mass and lifetime, because its trigger makes only modest requirements on transverse momentum.

## Detector description

The LHCb detector [15] is a single-arm forward spectrometer covering the pseudorapidity range $2<\eta <5$, designed for the study of particles containing $b$ or $c$ quarks. The detector includes a high-precision tracking system consisting of a silicon-strip vertex detector surrounding the $pp$ interaction region [16], a large-area silicon-strip detector located upstream of a dipole magnet with a bending power of about 4 Tm, and three stations of silicon-strip detectors and straw drift tubes [17] placed downstream of the magnet. The tracking system provides a measurement of momentum, $p$, with a relative uncertainty that varies from 0.4 % at low momentum to 0.6 % at 100 ${\mathrm {GeV/}\mathrm{c}}$. The minimum distance of a track to a primary vertex, the impact parameter, is measured with a resolution of $(15+29/p_\mathrm{T})\,\upmu \mathrm{m}$, where $p_\mathrm{T}$ is the component of $p$ transverse to the beam, in ${\mathrm {GeV/}\mathrm{c}}$. Different types of charged hadrons are distinguished using information from two ring-imaging Cherenkov detectors [18]. Photon, electron and hadron candidates are identified by a calorimeter system consisting of scintillating-pad and preshower detectors, an electromagnetic calorimeter and a hadronic calorimeter. Muons are identified by a system composed of alternating layers of iron and multiwire proportional chambers [19].

## Event simulation

For the event simulation, $pp$ collisions are generated using Pythia 6.4 [20] with a specific LHCb configuration [21] using CTEQ6L [22] parton density functions. Decays of hadronic particles are described by EvtGen  [23], in which final-state radiation is generated using Photos  [24]. The interaction of the generated particles with the detector and its response are implemented using the Geant4 toolkit [25, 26] as described in Ref. [27].

To simulate a signal event, a SM-like scalar Higgs boson with a mass of 120 ${\mathrm {GeV}/\mathrm{c}^2}$ is generated with Pythia through the gluon–gluon fusion mechanism, and is forced to decay into two spin-zero ${\pi _v}$ particles, each of which decays to $b\bar{b}$. Assuming the decay occurs via a vector or axial-vector coupling, the $b\bar{b}$ final state is preferred to light quarks, due to helicity conservation [6–8]. The average track multiplicity of the ${\pi _v}$ decay, including tracks from secondary $b$ and $c$ decays, varies from about 15 for a ${\pi _v}$ mass of 25 ${\mathrm {GeV}/\mathrm{c}^2}$ to about 20 for larger masses. Simulated events are retained if at least four charged tracks from the decay of the generated ${\pi _v}$ particles are within the LHCb acceptance, which corresponds to about 30 % of the cases. For ${\pi _v}$ particles within the acceptance on average about ten tracks can be reconstructed.

Simulated samples with ${\pi _v}$ lifetimes of 10$\mathrm{\,ps}$ and 100$\mathrm{\,ps}$ and ${\pi _v}$ masses of 25, 35, 43 and 50 ${\mathrm {GeV}/\mathrm{c}^2}$ are generated; other ${\pi _v}$ lifetimes are studied by reweighting these samples. Two additional samples are generated in which ${\pi _v}$ particles with a lifetime of 10$\mathrm{\,ps}$ and a mass of 35 ${\mathrm {GeV}/\mathrm{c}^2}$ decay to either $c\bar{c}$ or $s\bar{s}$ quark pairs.

## Event selection and signal extraction

The selection of candidates starts with the LHCb trigger [28], which consists of a hardware stage, based on information from the calorimeter and muon systems, followed by a software stage, which applies a full event reconstruction. The hardware trigger (L0) requires a single high-$p_\mathrm{T}$ hadron, electron, muon or photon signature. The thresholds range from $p_\mathrm{T} {}>1.48~{\mathrm {GeV/}\mathrm{c}}$ for muons, to transverse energy larger than $3.5\mathrm {\,Ge\,V}$ for hadrons. The total L0 efficiency, dominated by the hadron trigger selection, depends on the mass and final state of the ${\pi _v}$ particle and is typically 20 %, including the detector acceptance.

The software trigger is divided into two stages and consists of algorithms that run a simplified version of the offline track reconstruction, which allows identification of displaced tracks and vertices. For this analysis the primary signature in the first software stage (HLT1) is a single high-quality displaced track with high $p_\mathrm{T}$ . The efficiency of HLT1 relative to L0 accepted events is typically 60 %. However, this efficiency reduces rapidly for vertices that are displaced by more than about 5 mm from the beamline due to limitations in the track reconstruction in the vertex detector.

In the final trigger stage (HLT2) two different signatures are exploited. The first of these relies on the generic reconstruction of a displaced vertex, using an algorithm similar to that used for the primary vertex (PV) reconstruction [29]. Secondary vertices are distinguished from PVs using the distance to the interaction region in the transverse plane ($R_{xy}$). To eliminate contributions from interactions with material, a so-called ‘material veto’ removes vertices in a region defined as an envelope around the detector material [30]. Events are selected when they have a displaced vertex with at least four tracks, a sum of the scalar $p_\mathrm{T}$ of all tracks that is larger than 3 ${\mathrm {GeV/}\mathrm{c}}$, a distance $R_{xy}$ larger than 0.4 mm and an invariant mass of the particles associated with this vertex $m_\text {vtx}$ above 4.5 ${\mathrm {GeV}/\mathrm{c}^2}$. To further refine the selection, vertices are required to have either $R_{xy}> 2$ mm or $m_\text {vtx}> 10~{\mathrm {GeV}/\mathrm{c}^2}$.

The second HLT2 signature is designed to identify two-, three- and four-body exclusive $b$-hadron decays [31]. A multivariate algorithm is used for the identification of secondary vertices consistent with the decay of a $b$ hadron. The combined efficiency of the two HLT2 selections relative to events accepted by L0 and HLT1 is about 60 %.

The offline candidate reconstruction starts from a generic secondary vertex search, similar to that applied in the trigger, but using tracks from the offline reconstruction as input. At this stage at least six tracks per vertex are required and the sum of the scalar $p_\mathrm{T}$ of all tracks must be above 3 ${\mathrm {GeV/}\mathrm{c}}$. The vertex is required to have either $R_{xy}>0.4$ mm and $m_\text {vtx}> 9.7~{\mathrm {GeV}/\mathrm{c}^2}$, or $R_{xy}>2.5$ mm and $m_\text {vtx}> 8.5\,{\mathrm {GeV}/\mathrm{c}^2}$, or $R_{xy}> 4$ mm and $m_\text {vtx}> 6.5\,{\mathrm {GeV}/\mathrm{c}^2}$.

The vertex reconstruction is followed by a jet reconstruction procedure. Inputs to the jet clustering are obtained using a particle flow approach [32] that selects charged particles, neutral calorimeter deposits and a small contribution from $K_{s}^0$ and $\varLambda ^0$ decays. To reduce contamination from particles that do not originate from the displaced vertex, only charged particles that have a smaller distance of closest approach relative to the displaced vertex than to any PV in the event are retained. Furthermore, the distance to the displaced vertex is required to be less than 2 mm, which also allows tracks from displaced $b$ and $c$ vertices in the ${\pi _v} \rightarrow b\bar{b}$ decay chain to be accepted.

The jet clustering uses the anti-$k_\mathrm {T}$ algorithm [33] with a cone size of 0.7. Only jets with a $p_\mathrm{T}$ above 5 ${\mathrm {GeV/}\mathrm{c}}$ are used. Additional requirements are made to enhance the fraction of well-reconstructed hadronic jets: first, the charged particle with the largest $p_\mathrm{T}$ in the jet must have a $p_\mathrm{T}$ above 0.9 ${\mathrm {GeV/}\mathrm{c}}$, yet carry no more than 70 % of the $p_\mathrm{T}$ of the jet. Second, to remove jets whose energy is dominated by neutral particles, which cannot be unambiguously associated with a vertex, at least 10 % of the $p_\mathrm{T}$ of the jet must be carried by charged particles.

The di-jet invariant mass is computed from the reconstructed four-momenta of the two jets. Correction factors to the jet energy are determined from the simulation and parameterised as a function of the number of reconstructed PVs in the event, to account for effects due to multiple interactions and the underlying event [32].

Two further requirements are made to enhance signal purity. First, a corrected mass is computed as

1 $$\begin{aligned} m_\text {corr} \; = \; \sqrt{m^2 + \left( p \sin \theta \right) ^2} \, + \, p \sin \theta , \end{aligned}$$

where $m$ is the di-jet invariant mass and $\theta$ is the pointing angle between the di-jet momentum vector $\mathbf {p}$ and its displacement vector $\mathbf {d} = \mathbf {x}_\text {DV} - \mathbf {x}_\text {PV}$, where $\mathbf {x}_\text {DV}$ is the position of the displaced vertex and $\mathbf {x}_\text {PV}$ the position of the PV. To select candidates pointing back to a PV, only events with $m / m_\text {corr} > 0.7$ are retained. A requirement on this ratio is preferred over a requirement on the pointing angle itself, since its efficiency depends less strongly on the boost and the mass of the candidate.

Second, a requirement is made on the distance $\Delta R = \sqrt{\Delta \phi ^2 + \Delta \eta ^2}$ between the two jets, where $\phi$ is the azimuthal angle and $\eta$ the pseudorapidity. A background consisting of back-to-back jet candidates, for example di-jet $b\bar{b}$-events, appears mainly at large values of reconstructed mass, and is characterised by a large difference between the jets in both $\phi$ and $\eta$. Only candidates with $\Delta R < 2.2$ are accepted.

**Table 1:** Average number of selected candidates per event (efficiency) in % for the main stages of the offline selection for simulated $H^0 \rightarrow {\pi _v} {\pi _v}$ events with ${\pi _v} \rightarrow b\bar{b}$, $m_{H^0} =120\,{\mathrm {GeV}/\mathrm{c}^2}$, $m_{{\pi _v}} = 35\,{\mathrm {GeV}/\mathrm{c}^2}$ and $\tau _{{\pi _v}} =10\mathrm{\,ps}$. The pre-selection consists of the acceptance, trigger and offline vertex reconstruction. It represents the first stage in which the candidate yield on the total data sample, shown in the right column, can be counted. The reported uncertainty on the efficiency is only the statistical uncertainty from the finite sample size

Selection step	Signal efficiency	Yield in data
Pre-selection	2.125 $\pm$ 0.018	2,555,377
Jet reconstruction	1.207 $\pm$ 0.014	117,054
$m/m_\text {corr}$ and $\Delta R$	0.873 $\pm$ 0.012	58,163
Trigger on candidate	0.778 $\pm$ 0.012	29,921

**Table 2:** Average number of selected candidates per event (efficiency) in % for different ${\pi _v}$ masses, lifetimes and decay modes. The reported uncertainty is only the statistical uncertainty from the finite sample size. No simulated samples were generated for the 100$\mathrm{\,ps}$ decay to light quarks

Decay	$m_{{\pi _v}}$ [${\mathrm {GeV}/\mathrm{c}^2}$ ]	Signal efficiency	
		$\tau _{{\pi _v}} = 10\mathrm{\,ps}$	$\tau _{{\pi _v}} = 100\mathrm{\,ps}$
${\pi _v} \rightarrow b\bar{b}$	25	0.373 $\pm$ 0.008	0.0805 $\pm$ 0.0019
	35	0.778 $\pm$ 0.012	0.181 $\pm$ 0.005
	43	0.743 $\pm$ 0.011	0.183 $\pm$ 0.003
	50	0.573 $\pm$ 0.015	0.154 $\pm$ 0.004
${\pi _v} \rightarrow {{ c } {\overline{{ c }}}}$	35	2.18 $\pm$ 0.05	–
${\pi _v} \rightarrow {{ s } {\overline{{ s }}}}$	35	2.06 $\pm$ 0.04	–

Finally, in order to facilitate a reliable estimate of the trigger efficiency, only candidates triggered by particles belonging to one of the jets are kept. Table 1 shows the efficiency to select a ${\pi _v}$ particle, for an illustrative mass of 35 ${\mathrm {GeV}/\mathrm{c}^2}$ and lifetime of 10$\mathrm{\,ps}$, together with the yield in the data after the most important selection steps. The total efficiency for other masses and lifetimes, as well as for the decays to light quark jets, is shown in Table 2. The efficiencies listed in Tables 1 and 2 represent the number of selected candidates divided by the number of generated events. As the selection efficiencies for the two ${\pi _v}$ particles in an event are practically independent, the fraction of selected events with more than one candidate is less than a few percent in simulated signal. In data no events with more than one ${\pi _v}$ candidate are found.

**Fig. 1:**
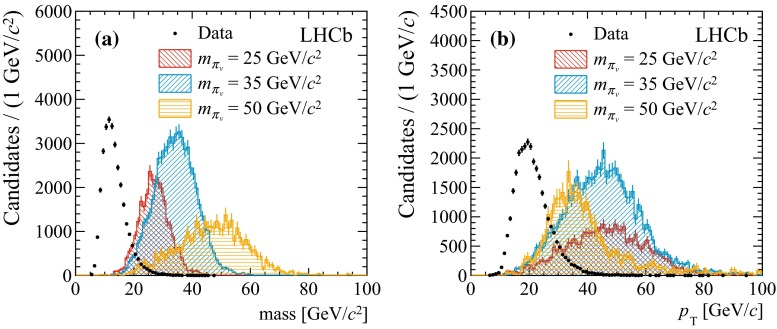
Invariant mass (**a**) and $p_\mathrm{T}$ distribution (**b**) for di-jet candidates in data and in hidden valley models with 25, 35 and 50 ${\mathrm {GeV}/\mathrm{c}^2}$ ${\pi _v}$ masses and 10$\mathrm{\,ps}$ lifetime. For visibility, the simulated signal is scaled to 0.62 $\text{ fb }^{-1}$ assuming a Higgs cross-section of 10 nb and branching fractions of 100 % for $\mathcal{B}(H\rightarrow {\pi _v} {\pi _v})$ and $\mathcal{B}({\pi _v} \rightarrow b\bar{b})$

**Fig. 2:**
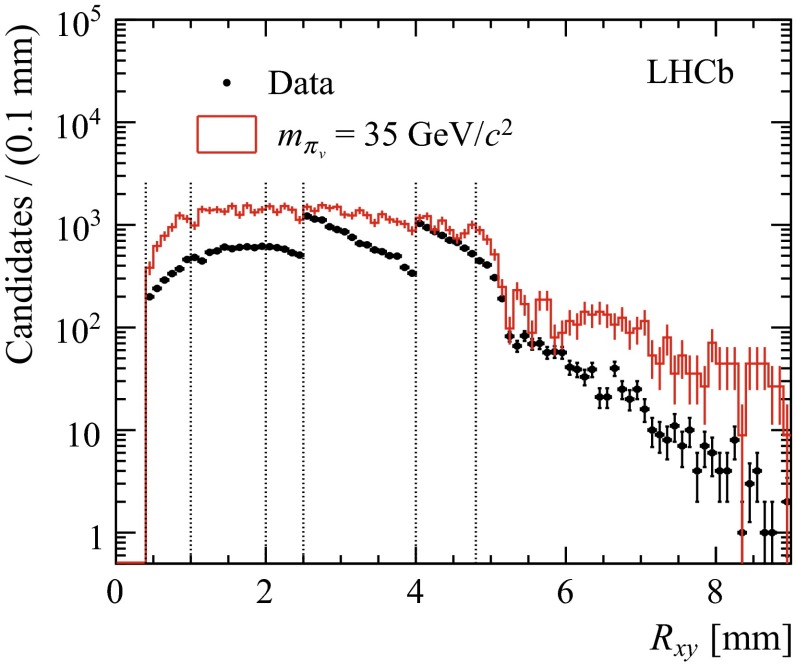
Distribution of the distance of the displaced vertex to the interaction region in the transverse plane for data and for a hidden valley model with $m_{{\pi _v}} = 35\,{\mathrm {GeV}/\mathrm{c}^2}$ and $\tau _{{\pi _v}} =10\mathrm{\,ps}$ after the full selection. For visibility, the simulated signal is scaled to 0.62 $\text{ fb }^{-1}$ assuming a Higgs cross-section of 10 nb and branching fractions of 100 % for $\mathcal{B}(H\rightarrow {\pi _v} {\pi _v})$ and $\mathcal{B}({\pi _v} \rightarrow b\bar{b})$. The boundaries of the intervals used in the fit are indicated by the dotted lines. The generated $R_{xy}$ distribution is approximately exponential with an average of about 2 mm

Figure 1 shows the mass and $p_\mathrm{T}$ distributions for selected di-jet candidates in data and in simulated signal events, assuming a ${\pi _v}$ particle with a mass of 25, 35 or 50 ${\mathrm {GeV}/\mathrm{c}^2}$. The turn-on at low values in the mass distribution of events observed in data (Fig. 1a) is caused by the minimum $p_\mathrm{T}$ requirement on the jets. The rest of the distribution falls off exponentially. The $p_\mathrm{T}$ distribution shown in Fig. 1b illustrates that long-lived particles with a higher mass have lower $p_\mathrm{T}$ as there is less momentum available in the Higgs decay. This affects the selection efficiency since for a given decay time the transverse decay length is proportional to $p_\mathrm{T}$.

Studies on simulated events have shown that both the shape and the normalisation of the mass distribution in data are compatible with the expected background from $b\bar{b}$ production. It is not possible to generate sufficiently large samples of $b\bar{b}$ events to use these for a quantitative estimate of the background after the final selection. Therefore, the signal yield is extracted by a fit to the invariant mass distribution assuming a smooth shape for the background, as discussed in Sect. 6.

Since the background yield, the shape of the background invariant mass distribution and the selection efficiency strongly depend on the radial displacement $R_{xy}$, limits are extracted from a simultaneous maximum likelihood fit to the di-jet invariant mass distribution in five bins of $R_{xy}$. The intervals are chosen in the most sensitive region, between 0.4 and 4.8 mm. The events at larger radii are not used as they contribute only marginally to the sensitivity. Figure 2 shows the distribution of $R_{xy}$ of selected displaced vertices for data and simulated signal events, together with the bin boundaries. The effect of the reduction in efficiency at large radii due to the material veto and the HLT1 trigger is visible, as is the effect of requirements on $R_{xy}$ in the trigger. The trigger effects are more pronounced in data than in simulated signal, because signal events are less affected by cuts on the vertex invariant mass.

**Fig. 3:**
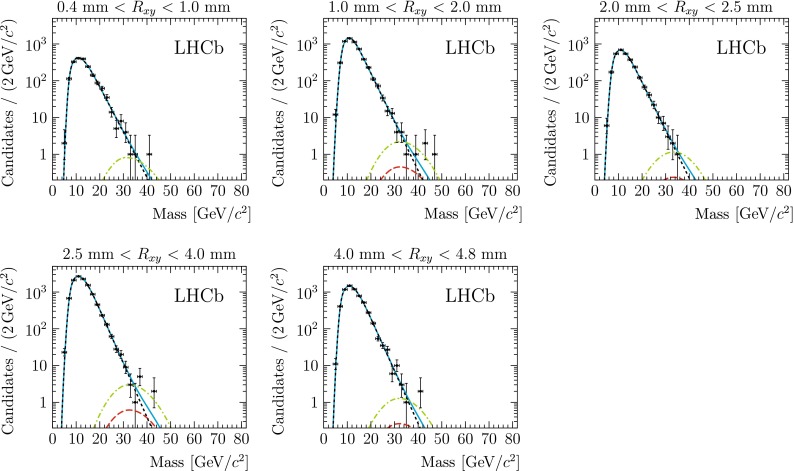
Di-jet invariant mass distributions for each of the five $R_{xy}$ bins, superimposed with the fits for a hidden valley model with $m_{{\pi _v}} = 35\,{\mathrm {GeV}/\mathrm{c}^2}$ and $\tau _{{\pi _v}} =10\mathrm{\,ps}$. The *blue line* indicates the result of the total fit to the data. The *black short-dashed line* is the background-only contribution, and the *red long-dashed line* is the fitted signal contribution. For illustration, the *green dash-dotted line* shows the signal scaled to a cross-section of 17 pb, which corresponds to the SM Higgs production cross-section at 7 TeV [35]

The background di-jet invariant mass distribution is characterised by an exponential falloff, with a low-mass threshold determined mostly by the minimum $p_\mathrm{T}$ requirement of the jets. It is modelled by a single-sided exponential function convoluted with a bifurcated Gaussian function. The parameters of the background model are fitted to data, independently in each $R_{xy}$ bin. The signal is modelled by a bifurcated Gaussian function, whose parameters are determined from simulated events in bins of $R_{xy}$. The effect of the uncertainty on the jet-energy scale is included by a scale parameter for the mass, which is common to all bins and constrained using a sample of $Z + \text {jet}$ events, as explained in Sect. 5. Additional nuisance parameters are added to account for the finite statistics of the simulated samples and the systematic uncertainties on the signal efficiency and the luminosity. The fit model is implemented using the RooFit  [34] package. Figure 3 shows the fit result in the five radial bins for a signal model with $m_{{\pi _v}} = 35\,{\mathrm {GeV}/\mathrm{c}^2}$ and $\tau _{{\pi _v}} = 10\mathrm{\,ps}$.

## Systematic uncertainties

Several sources of systematic uncertainties have been considered. The uncertainties depend on the ${\pi _v}$ mass and are summarised in Table 3. The uncertainty on the vertex finding efficiency is assessed by comparing the efficiency of the vertexing algorithm on a sample of $B^{0}\rightarrow j/\psi K^{*0}$ with $K^{*0}\rightarrow K^{+}{{\pi } ^-}$ events in data and simulation as a function of $R_{xy}$. The efficiency difference is about 7.5 % at large $R_{xy}$, which is taken as an estimate of the uncertainty on the vertex finding algorithm efficiency. Since the ${\mathrm {B}} ^0$ vertices have only four tracks, and the ${\pi _v}$ decays studied in this paper have typically more tracks, this is considered a conservative estimate. The uncertainty on the track finding efficiency for prompt tracks in LHCb is 1.4 % per track, with a small dependence on track kinematics [36]. The uncertainty for displaced tracks was evaluated in the context of a recent LHCb measurement of $b$-hadron lifetimes [37] and extrapolated to larger $R_{xy}$, leading to a per-track uncertainty of 2 %. Due to requirements on the minimal number of tracks in the vertex, this translates into an uncertainty on the vertex finding efficiency, which is estimated to be 2 % for signal events. Adding in quadrature the track efficiency and the vertex finding algorithm efficiency uncertainties leads to a total uncertainty of 7.9 % on the vertex reconstruction. The selection on the vertex sum-$p_\mathrm{T}$ and mass is affected by the track finding efficiency as well. Propagating the per-track uncertainty leads to an uncertainty on the vertex selection efficiency of up to 2.9 %, depending on the ${\pi _v}$ mass.

**Table 3:** Systematic uncertainties on the selection efficiency and luminosity for simulated hidden valley events with a lifetime of 10$\mathrm{\,ps}$ and various ${\pi _v}$ masses

Source	Relative uncertainty (%)			
${\pi _v}$ Mass [${\mathrm {GeV}/\mathrm{c}^2}$ ]	25	35	43	50
Vertex reconstruction	7.9	7.9	7.9	7.9
Vertex scalar-$p_\mathrm{T}$ and mass	2.9	2.3	2.0	1.7
Jet reconstruction	1.3	0.6	0.4	0.3
Jet identification	2.9	3.0	3.2	3.2
Jet pointing	4.6	2.9	2.6	2.0
L0 trigger	4.6	4.5	4.5	4.4
HLT1 trigger	4.1	4.0	4.0	4.3
HLT2 trigger	5.9	5.9	6.1	6.3
Luminosity	1.7	1.7	1.7	1.7
Total	13.3	12.7	12.6	12.6

**Table 4:** Observed 95 % CL cross-section upper limits on $\sigma (H)\times \mathcal{B}(H\rightarrow {\pi _v} {\pi _v})$ (in $\mathrm \,pb$) on a hidden valley [6–8] model for various ${\pi _v}$ masses and lifetimes. Both ${\pi _v}$ particles are assumed to decay into ${\mathrm {b}} {\overline{{\mathrm {b}}}}$, unless specified otherwise

${\pi _v}$ Mass [${\mathrm {GeV}/\mathrm{c}^2}$ ]	${\pi _v}$ Lifetime [$\mathrm{\,ps}$ ]							
	1	2	5	10	20	50	100	200
25	106.3	54.6	43.8	54.2	80.0	164.1	285.7	588.5
35	19.0	10.4	8.0	8.9	13.3	25.4	46.5	89.8
43	10.5	5.6	4.4	4.7	6.7	12.4	22.7	42.8
50	10.6	5.1	3.7	3.8	4.8	9.3	16.2	29.3
35 (${\pi _v} \rightarrow c\bar{c}$)	3.7	2.4	2.1	2.4	3.4	6.7	12.5	24.1
35 (${\pi _v} \rightarrow s\bar{s}$)	3.4	2.1	1.9	2.2	3.3	6.4	11.6	22.0

The uncertainties related to the jet selection are determined by comparing jets in data and simulation on a sample of $$Z + \text {jet}$$ events, analogously to a recent LHCb measurement of $$Z + \text {jet}$$ production [32]. The $$\mathrm {Z}$$ candidate is reconstructed in the $$\mu ^+$$$$\mu ^-$$ final state from two oppositely charged tracks, identified as muons, that form a good vertex and have an invariant mass in the range 60–120 GeV/c$$^2$$. Jets are reconstructed using the same selection of input particles as in the reconstruction of jets for long-lived particles, except that the origin vertex is in this case the PV consistent with the $$Z$$ vertex. The differences between data and simulation in the $$Z + \text {jet}$$ sample are parameterised as function of the jet $$p_\mathrm{T}$$ and subsequently propagated to the simulated hidden valley signal samples.

The uncertainty on the jet energy scale is derived from the ratio of transverse momenta of the jet and the $Z$, which are expected to have a back-to-back topology, and correlated transverse momenta. Data and simulation agree within about 2 %, resulting in an uncertainty on the di-jet invariant mass scale of 4 %. This uncertainty on the signal shape is taken into account in the fitting procedure. The uncertainty on the jet-energy scale also affects the jet reconstruction efficiency due to the requirement on the minimum jet $p_\mathrm{T}$. It leads to an uncertainty on the efficiency between 0.3 and 1.3 %, depending on the assumed ${\pi _v}$ particle mass. The uncertainty on the hadronic jet identification requirements are assessed using the $Z + \text {jet}$ sample as well and amount to about 3 %.

The resolutions on the pointing angle $\theta$ and on $\Delta R$ are dominated by the resolution on the direction of the ${\pi _v}$ candidate, which in turn is determined by the jet angular resolution. The latter is estimated from the difference between data and simulation in the resolution of the azimuthal angle between the jet and the $Z$. Due to the limited statistics in the $Z + \text {jet}$ sample a relatively large uncertainty between 2.0 and 4.6 % is obtained, depending on the ${\pi _v}$ mass.

The trigger selection efficiency on signal is determined from the simulation. The trigger efficiencies in data and simulation are compared using a sample of generic $B\rightarrow J/\psi X$ events that contain an offline reconstructed displaced vertex, but are triggered independently of the displaced vertex trigger lines. The integrated efficiency difference for the trigger stages L0, HLT1 and HLT2 amounts to systematic uncertainties of at most 4.6, 4.3 and 6.3 % respectively. This is a conservative estimate since the trigger efficiencies for the sample of displaced $J/\psi$ vertices are smaller than the efficiencies for the signal, which consists of heavier, more displaced objects with a larger number of tracks. Finally, the uncertainty on the luminosity at the LHCb interaction point is 1.7 % [38].

Several alternatives have been considered for the background mass model, in particular with an additional exponential component, or a component that is independent of the mass. With these models the estimated background yield at higher mass is larger than with the nominal background model, leading to tighter limits on the signal. As the nominal model gives the most conservative limit, no additional systematic uncertainty is assigned.

## Results

The fit procedure is performed for a ${\pi _v}$ mass of 25, 35, 43 and 50 ${\mathrm {GeV}/\mathrm{c}^2}$ and for several values of the lifetime in between 1 and 200$\mathrm{\,ps}$. No significant signal is observed for any combination of ${\pi _v}$ mass and lifetime. Upper limits are extracted using the $\text {CL}_\text {s}$ method [39] with a frequentist treatment of the nuisance parameters described above, as implemented in the RooStats  [40] package.

**Fig. 4:**
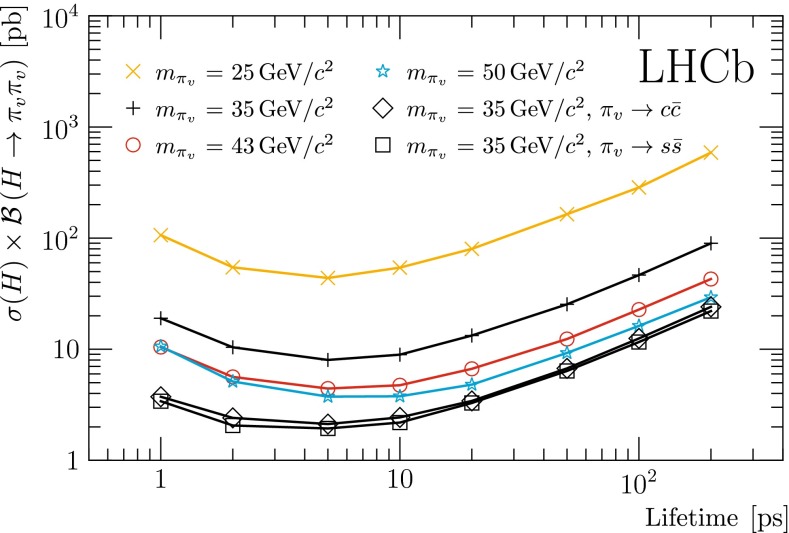
Observed 95 % CL cross-section upper limits on a hidden valley model [6–8] for various ${\pi _v}$ masses, as a function of ${\pi _v}$ lifetime. Both ${\pi _v}$ particles are assumed to decay into $b\bar{b}$, unless specified otherwise

Limits are set on the Higgs production cross-section multiplied by the branching fraction into long-lived particles $\sigma (H)\times \mathcal{B}(H\rightarrow {\pi _v} {\pi _v})$. In the simulation it is assumed that both ${\pi _v}$ particles decay to the same final state. If the decay width of the ${\pi _v}$ particle is dominated by final states other than $q\bar{q}$, the limits scale as $1/(\mathcal{B}_{q\bar{q}}(2-\mathcal{B}_{q\bar{q}}))$ where $\mathcal{B}_{q\bar{q}}$ is the ${\pi _v} \rightarrow q\bar{q}$ branching fraction. The obtained 95 % CL upper limits on $\sigma (H)\times \mathcal{B}(H\rightarrow {\pi _v} {\pi _v})$, under the assumption of a 100 % branching fraction to $b\bar{b}$, are shown in Table 4 and in Fig. 4. As the background decreases with the observed di-jet invariant mass, the limits become stronger with increasing ${\pi _v}$ mass. The sensitivity has an optimal value at a lifetime of about 5$\mathrm{\,ps}$.

Additional limits are set on models with a ${\pi _v}$ particle decaying to $c\bar{c}$ and to $s\bar{s}$. The limits for ${\pi _v}$ decay to $u\bar{u}$ and $d\bar{d}$ are expected to be the same as for $s\bar{s}$. The light quark decays result in a higher displaced vertex track multiplicity, and lighter jets, leading to a higher selection efficiency. Consequently, the limits for decays to light quark jets are more stringent than those for decays to $b$-quark jets.

## Conclusion

A search has been presented for massive, long-lived particles in a sample of $pp$ collisions at $\sqrt{s}=7$ TeV, corresponding to an integrated luminosity of 0.62 $\text{ fb }^{-1}$, collected by the LHCb experiment. The long-lived spin-zero particles are assumed to be pair-produced in the decay of a 120 ${\mathrm {GeV}/\mathrm{c}^2}$ SM Higgs, and to decay to two hadronic jets. They appear for instance as ${\pi _v}$ particles in hidden valley models. A single ${\pi _v}$ particle is identified by a displaced vertex and two associated jets. No significant signal for ${\pi _v}$ particles with a mass between 25 and 50 ${\mathrm {GeV}/\mathrm{c}^2}$ and a lifetime between 1 and 200$\mathrm{\,ps}$ is observed. Assuming a 100% branching fraction to $b$-quark jets, the 95 % CL upper limits on the production cross-section $\sigma (H)\,\times \,\mathcal{B}(H\rightarrow {\pi _v} {\pi _v})$ are in the range 4–600 pb.

The results cover a region in mass and lifetime that so far has been unexplored at the LHC. The obtained upper limits are more restrictive than results from the Tevatron experiments in the same mass and lifetime region. The best sensitivity is obtained for ${\pi _v}$ particles with a lifetime of about 5$\mathrm{\,ps}$ and a mass above approximately 40 ${\mathrm {GeV}/\mathrm{c}^2}$. The SM Higgs cross-section at 7 TeV is about 17 pb [35]. The measurements in the most sensitive region exclude branching fractions of greater than 25 % for a SM Higgs boson to pair produce ${\pi _v}$ particles that decay to two hadronic jets.

## Footnotes

1 The results are equally valid for a Higgs particle with a mass up to 126 ${\mathrm {GeV}/\mathrm{c}^2}$ within a few percent.
